# Weight Stigma among Young Adults in Thailand: Reliability, Validation, and Measurement Invariance of the Thai-Translated Weight Self Stigma Questionnaire and Perceived Weight Stigma Scale

**DOI:** 10.3390/ijerph192315868

**Published:** 2022-11-29

**Authors:** Paratthakonkun Chirawat, Ruckwongpatr Kamolthip, Rattana Rattaprach, Siti R. Nadhiroh, Serene En Hui Tung, Wan Ying Gan, Meephiam Pinyo, Teosagul Nabpran, Kaitlin N. Rozzell-Voss, Janet D. Latner, Chung-Ying Lin

**Affiliations:** 1 College of Sports Science and Technology, Mahidol University, Sala Ya, Phutthamonthon, Nakhon Pathom 73170, Thailand; 2 Institute of Allied Health Sciences, National Cheng Kung University Hospital, College of Medicine, National Cheng Kung University, Tainan 70142, Taiwan; 3 Department of Nutrition, Faculty of Public Health, Universitas Airlangga, Surabaya 60115, Indonesia; 4 Division of Nutrition and Dietetics, School of Health Sciences, International Medical University, Kuala Lumpur 57000, Malaysia; 5 Department of Nutrition, Faculty of Medicine and Health Sciences, Universiti Putra Malaysia, Serdang 43400, Malaysia; 6 Department of Forensic Science, Royal Police Cadet Academy, Nakhon Pathom 73110, Thailand; 7 Faculty of Humanities and Social Sciences, Songkhla Rajabhat University, Songkhla 90000, Thailand; 8 Department of Psychology, University of Hawaii at Manoa, Honolulu, HI 96822, USA; 9 Biostatistics Consulting Center, National Cheng Kung University Hospital, College of Medicine, National Cheng Kung University, Tainan 70142, Taiwan; 10 Department of Occupational Therapy, College of Medicine, National Cheng Kung University, Tainan 70142, Taiwan; 11 Department of Public Health, College of Medicine, National Cheng Kung University, Tainan 70142, Taiwan

**Keywords:** measurement invariance, psychometrics, obesity, weight stigma, young adults

## Abstract

The previous studies found that the Weight Self Stigma Questionnaire (WSSQ) and Perceived Weight Stigma Scale (PWSS) have shown well-established psychometric properties for measuring weight stigma with strong reliability and validity from different languages. However, there is a lack of an appropriate instrument in assessing weight stigma in Thai samples. This study aimed to examine the Thai WSSQ and PWSS among Thai university students. Both instruments were also assessed for their measurement invariance across gender and weight status subgroups. A cross-sectional study was conducted on 801 university students in Thailand between January 2022 and July 2022. All participants completed a demographic questionnaire and a Thai version of the WSSQ, PWSS, and Depression Anxiety Stress Scale-21 (DASS-21) via an online survey. Reliability, validity, measurement invariance, and correlational analyses were performed to investigate whether the Thai versions of the WSSQ and PWSS psychometric properties were acceptable. Both translated questionnaires demonstrated overall acceptable psychometric properties and revealed a two-dimensional structure for the WSSQ, and unidimensional structure for the PWSS. Measurement invariance was obtained across gender and weight status subgroups. Additionally, both translated WSSQ and PWSS were significantly correlated with DASS-21. The Thai-translated WSSQ and PWSS showed strong validity, reliability, and factorial invariance across different subgroups for measuring weight stigma among Thai university students.

## 1. Introduction

Many studies have shown that people with overweight are more likely to experience negative health consequences (i.e., physiological, psychological, and social problems) [1,2]. Individuals with overweight are likely to experience or encounter weight bias (specifically, weight-based discrimination) in their daily lives across different environments (e.g., workplace, medical) and various relationships (i.e., family, peers, and colleagues) [3,4]. Weight-related self-stigma has been well-documented in many studies as the development of individuals who have self-devaluation due to perceiving the negative bias of their weight status [2,3,4]. Furthermore, individuals could experience weight stigma from others’ negative attitudes and beliefs which influence others to expose individuals who are different to the norm, i.e., people with overweight, to three components (i.e., stereotype, prejudice, and discrimination) [1,2,3]. Moreover, those with overweight are faced with negative trait-based stereotypes associated with weight (e.g., ugly, lazy), which may expose them to prejudice and discrimination [5,6]. Evidence indicates that weight stigma impacts a variety of health behaviors, in particular, it can increase eating disturbances (i.e., emotional eating) [7]. Nevertheless, even individuals who do not have overweight or obesity, but perceive themselves as overweight, may encounter problems with weight stigma and body-related emotions (e.g., shame and blame) [8]. Therefore, individuals across the weight spectrum who experience weight stigma are likely to experience negative health consequences and impaired quality of life. 

Previous research indicated that Thai young adults perceived beauty standards from cultural ideals, resulting in psychological distress (e.g., depression, body dissatisfaction, and low self-confidence) and disordered eating behaviors such as self-imposed vomiting and dietary control [9,10]. Notably, Thailand has witnessed an increased rate of overweight among adults from 2012 to 2018, with the mean body mass index (BMI) changing from 23.9 to 25.0 kg/m^2^ [11,12]. Moreover, a study reported that underweight Asian university students (including Thailand) were prone to perceive themselves as having a higher weight status than they actually did, which led to greater symptoms of psychopathology, including eating disturbances [13]. Similarly, a previous study among Thai university students reported that those with overweight were at higher risk of psychological distress (i.e., depression) due to trying to lose or control their weight [14]. The literature indicated that misperceived weight status could be a major factor in ideal body type among Thai young adults, specifically, in people with underweight [14]. Additionally, past findings have highlighted that a slim figure is perceived as most attractive among Thai young adults [14]. Therefore, we speculate that young Thai people might encounter weight stigma and its negative consequences, regardless of their weight status. Despite the negative effects of weight stigma, there is still a lack of research in this domain among Thai people, in addition to a lack of psychometric data to ensure an appropriate measure of weight stigma. 

Having accurate and appropriate instruments to measure weight stigma could benefit healthcare services because such instruments may help healthcare providers and related stakeholders understand the scope and impact of weight stigma. The Weight Self-Stigma Questionnaire (WSSQ) is a commonly used instrument to assess weight stigma using a two-factor structure including self-devaluation and the fear of enacted stigma [4,6]. The WSSQ was developed by Lillis et al. [4] to assess and comprehend the several aspects of the internalized weight stigma. The researchers extended the assessment to the nature of weight-related self-stigma including experiencing social prejudice (i.e., enacted stigma) and also concerning oneself with others’ internalized weight stigma (i.e., fear of enacted stigma) [4]. The scholars believed that WSSQ would show a reliable and consistent measurement to assess the internalized weight stigma and sensitivity to various negative health outcomes (e.g., psychological distress, eating disturbances) [4]. The internal consistency of the WSSQ was found to be promising in many languages across different populations [2,15,16], for instance, among Iranian-speaking people with overweight and obesity, German-speaking people with severe obesity, and French-speaking adolescents with overweight and obesity [2,15,16].

The Perceived Weight Stigma Scale (PWSS) assesses another aspect of weight bias: the perception of weight stigma [17]. The PWSS was developed by Schafer and Ferraro to assess the perceptions of weight discrimination [18]. The PWSS was developed to assess the perceptions of weight discrimination [18]. The researchers believed that people could perceive weight stigma and influence them as being overweight, and, in addition to those with perceived weight stigma, could impact negative health outcomes [19]. Therefore, assessing perceived weight stigma could explain how weight stigma influences people’s health. Similar to the WSSQ, the internal consistency of PWSS was found to be promising across different populations [18]. For example, the measure was validated among Chinese-speaking children and young adults and Malaysian university students [6,17,20]. To our knowledge, no studies have validated the WSSQ and PWSS in a Thai sample. Therefore, we expect that the WSSQ and PWSS in Thai-translated versions will be useful for Thai healthcare professionals, and in addition, these translated instruments will show evidence of adequate psychometric properties.

Moreover, the present study intended to establish measurement invariance of the Thai WSSQ and PWSS across subgroups (i.e., genders (male vs. female), and weight status (overweight vs. non-overweight)). Measurement invariance is generally conducted to compare different populations, culture, and languages in a questionnaire [2,21]. Additionally, previous studies indicated a need for factorial invariance across subgroups to strengthen the instrument [21,22]. Accordingly, we performed measurement invariance to examine the structure and verify the invariance of the WSSQ and PWSS across gender and weight status subgroups.

We hypothesized that both the Thai WSSQ and Thai PWSS would have satisfactory psychometric properties and would be measurement invariant across all subgroups. Validating these scales may be useful and crucial for healthcare providers and researchers for their use in Thailand.

## 2. Materials and Methods

### 2.1. Participants

This study obtained approval from Mahidol University Central Institutional Review Board (MU-MOU COA 2022/006.2001) before data collection. Our study was conducted from January 2022 to July 2022. A total of 801 participants were recruited through convenience sampling from university students in Thailand. There were slightly over two-thirds female (66.9%), and most participants had no chronic condition or diseases (91.5%). The mean age of the participants was 20.69 ± 3.7 years (Table A1).

Participants were given an online questionnaire which research assistants disseminated through the university website, Facebook, and posters around campus. Participants accessed a QR code to log onto a *Google form* and were then required to provide e-form informed consent on the first page of the online survey. All participants completed the demographic questionnaire, and the WSSQ, PWSS, and DASS-21. The inclusion criteria comprised: (1) studying in a university in Thailand (including undergraduates and postgraduates); (2) an understanding of Thai language; (3) age ≥ 18 years.

Thai translation was conducted according to a standard translation procedure [23]. In brief, the WSSQ and PWSS were separately translated into Thai by two Thai bilingual scholars in sports science and psychology. One forward translation was then conducted by two independent linguists for backward translation into English. Subsequently, one forward and two backward translations were carried out by a panel including three experts (i.e., a psychologist, a psychiatrist, and a public health expert) to guarantee conceptual equivalency. Finally, these three experts decided the most appropriate Thai-translated version of the WSSQ and PWSS.

### 2.2. Measures

#### 2.2.1. Demographic and Anthropometric Measures

Participants were asked for their age, gender, marital status, degree of study, self-reported weight (in kilograms), self-reported height (in centimeters), and if they had any health conditions or chronic diseases. According to the Asian standard, the BMI was used to categorize weight status (i.e., <23 kg/m^2^ as non-overweight, and >23 kg/m^2^ as overweight) into two groups including non-overweight (68.16%), and overweight (31.84%) participants [24]. The mean BMI was 21.19 ± 1.77 kg/m^2^ (Table A1).

#### 2.2.2. Weight Self-Stigma Questionnaire (WSSQ)

The WSSQ is a self-reported instrument assessing weight-related self-stigma [25]. It contains 12 items, and every item is rated on a 5-Likert point scale (1 = *strongly disagree* to 5 = *strongly agree*) [25]. The WSSQ is comprised of two subscales including self-devaluation (factor I, items 1–6) and fear of enacted stigma (factor II, items 7–12) [4,15]. An example of self-devaluation item is “I’ll always go back to being overweight” and an example item for the fear of enacted stigma subscale is “I feel insecure about others’ opinions of me”. Total scores are calculated by summing all 12 items, with higher scores indicating greater weight-related self-stigma. A previous study indicated that the internal consistency of WSSQ was acceptable in English (Cronbach’s α = 0.88 for full scale; α = 0.81 for self-devaluation subscale; α = 0.87 for fear of enacted stigma subscale) [4].

#### 2.2.3. Perceived Weight Stigma Scale (PWSS)

The PWSS is a self-report instrument intended to assess perceived weight stigma which is based on participants’ weight perception [20]. It contains 10 items, and every item is rated on a dichotomous scale (1 = yes and 0 = no) [20]. An example item for PWSS is “People behave as if you are inferior because of your weight status”. Total scores are calculated by summing all items. Higher scores indicate greater perceived weight stigma. The internal consistency of the PWSS was shown to be acceptable in English (Cronbach’s α = 0.83) and Chinese (Cronbach’s α = 0.84) in past studies [17,20].

#### 2.2.4. Depression Anxiety Stress Scale-21 (DASS-21)

The DASS-21 is a self-report instrument assessing depression, anxiety, and stress [26,27]. It contains 21 items, and each item is rated on a 4-Likert point scale (0 = *did not apply to me at all* to 3 = *applied to me very much or most of the time*). The DASS-21 has three subscales: depression, anxiety, and stress. Each subscale has seven items [28]. An example item for the depression subscale is “I couldn’t seem to experience any positive feeling at all”; for the anxiety subscale, “I was aware of dryness of my mouth”; and for the stress subscale, “I found it hard to wind down”. Total scores are calculated by summing all 21 items [27]. The internal consistency of DASS-21 was acceptable in English (Cronbach’s α = 0.93 for the full scale; α = 0.88 for the depression subscale; α = 0.82 for the anxiety subscale; α = 0.9 for the stress subscale) and Thai (Cronbach’s α = 0.90 for full scale; α = 0.80 for depression subscale; α = 0.73 for anxiety subscale; α = 0.80 for stress subscale) [29,30].

Additionally, we performed the DASS-21 instrument as a validation criterion for WSSQ and PWSS, given that the previous study reported that DASS-21 had presented potential and reliable psychometric properties to assess emotional distress among Asian populations [26]. Moreover, evidence found that DASS-21 could assess the psychological distress across cultures [26]. Therefore, we established DASS-21 as the validation indicator between WSSQ and PWSS to assess psychological issues related to mental health problems.

### 2.3. Statistical Analysis

This study was performed using the JASP version 0.16.3 to analyze all data including descriptive statistics, reliability analysis, item analysis, fit indices, and measurement invariance across group (i.e., gender and weight status) in the WSSQ and PWSS. Confirmatory Factor Analysis (CFA) was used to verify the factor structure of the WSSQ and PWSS, and Pearson correlations were used to assess their relationship with other variables in the study [31].

Descriptive analyses were used to examine the participants’ demographic makeup, and mean scores on the WSSQ, PWSS, and DASS-21. Additionally, item distributions were tested using skewness and kurtosis. For the WSSQ, skewness ranged between −1.10 and −0.12 in factor I; and between −0.17 and 0.68 in factor II. Similarly, kurtosis ranged between −0.93 and 1.48 in factor I; and between −0.51 and −0.84 in factor II. For the PWSS, skewness ranged from 1.30 and 4.27; and kurtosis ranged from −0.32 and 16.24. 

Next, CFA was used to examine whether the WSSQ is a two-factor structure, and the PWSS is a unidimensional structure. All items of the WSSQ and PWSS were investigated using factor loadings from CFA and the corrected item–total correlation. A diagonally weighted least square (DWLS) estimator was used in the CFA because of the ordinal/dichotomous scales used in the WSSQ and PWSS [32,33]. The reliability was investigated using Cronbach’s α and McDonald’s ω with values (in α and ω) greater than 0.7 indicating acceptable internal consistency [34,35].

According to prior evidence, Cronbach’s α was used to report internal consistency [34]. However, some researchers argued that Cronbach’s α has the limitation of tau-equivalence and also suggested using McDonald’s ω instead of Cronbach’s α to report the internal consistency [35]. Therefore, we decided to report Cronbach’s α to link up with prior research and also using McDonald’s ω to tackle the problem of tau-equivalence in Cronbach’s α [35].

To investigate the model fit, a χ^2^ test, in addition to descriptive fit indices (comparative fit index (CFI), a Tucker–Lewis index (TLI), a root mean square error of approximation (RMSEA), and a standardized root mean square residual (SRMR)) were used. Specifically, a nonsignificant χ^2^ and the values of both CFI and TLI above 0.9, and both RMSEA and SRMR below 0.08 were used together to decide if model fit is satisfactory [36,37]. Standardized factor loadings above 0.4 were deemed acceptable measures of the constructs [38]. However, we did not use the chi-square difference test to assess better fit index model, although, the nonsignificant chi-square difference test is preferred. The previous study suggested that performing chi-square statistics has limitations which are sensitive with a larger sample size [39]. The researchers encouraged the report and assess combination including chi-square, RMSEA, CFI, and SRMSR to consider model fit indices [39].

For measurement invariance, multi-group CFA (MGCFA) was performed to analyze invariance across groups. We separately categorized two groups for gender (female and male) and weight status (non-overweight and overweight). Three nested CFA models (i.e., configural model, a metric invariance model with factor loadings constrained equally across groups, and a scalar invariance model with factor loadings and items’ intercepts constrained equally across groups) were used to investigate the measurement invariance across groups in factorial structures [2,6,40]. The measurement invariance across subgroup (by gender or by weight status) of WSSQ and PWSS structures would be supported with a nonsignificant *χ*^2^ difference test, and all values with comparison of ∆CFI, ∆RMSEA, ∆SRMR below 0.01 between each nested model [2]. Lastly, Pearson correlations were used to analyze the associations between WSSQ, PWSS, DASS-21, and BMI.

## 3. Results

According to our findings, we found the high correlations of WSSQ presented between the entire scale and both two subscales, r = 0.85 to 0.90 (*p* < 0.001). Additionally, both two subscales of WSSQ showed the moderate correlation, r = 0.55 (*p* < 0.001).

As reported in Table 1 and Table 2, CFA results showed that the WSSQ has a two-factor structure, and the PWSS has a unidimensional structure. The psychometric properties of both the WSSQ and PWSS were additionally supported by CFA results showing good fit indices (except for significant χ^2^ due to large sample size). For WSSQ, CFI = 0.969; TLI = 0.961; RMSEA = 0.072; and SRMR = 0.073. Similarly, for PWSS, *p*-value of χ^2^ = 0.122; CFI = 0.995; TLI = 0.994; RMSEA = 0.019; and SRMR = 0.052. The standardized factor loadings were adequate for WSSQ (factor I = 0.48–78; factor II = 0.59–0.82) with satisfactory item-total correlation (factor I = 0.51–0.69; factor II = 0.45–0.76). The standardized factor loadings were also adequate for PWSS items (0.48–0.68), with satisfactory item-total correlation (0.46–0.62).

Additionally, the internal consistency was acceptable for the entire WSSQ (both Cronbach’s α and McDonald’s ω = 0.88), its two factors (factor I: both Cronbach’s α and McDonald’s ω = 0.83; factor II: both Cronbach’s α and McDonald’s ω = 0.86), and the PWSS (both Cronbach’s α and McDonald’s ω = 0.85).

As reported in Table 3, the three nested models presented that both self-report measures showed invariance across weight and gender groups. For the WSSQ, the factor-loading constrained models showed nonsignificant χ^2^ difference from the configural models across the gender group (∆*χ*^2^ = 15.57 (*p* = 0.113)) and the factor-loading constrained models showed significant χ^2^ difference from the configural models across the weight status group (∆*χ*^2^ = 23.63 (*p* = 0.009)). The factor-loading constrained models and items-intercept constrained models showed significant χ^2^ difference from the configural models across both gender and weight status groups (∆*χ*^2^ = 34.65 (*p* = 0.0001) and 68.39 (*p* < 0.001)). However, all values of ∆CFI, ∆RMSEA, and ∆SRMR were below 0.01, which indicated measurement invariance of the WSSQ structure was supported across the gender and weight status groups.

For the PWSS (Table 3), the factor-loading constrained models showed significant χ^2^ difference from the configural models across the gender group (∆*χ*^2^ = 20.51 (*p* = 0.015)) and nonsignificant χ^2^ difference from the configural models across the weight status group (∆*χ*^2^ = 6.47 (*p* = 0.692)). The factor-loading constrained models and items-intercept constrained models showed significant χ^2^ difference from the configural models across the gender group (∆*χ*^2^ = 18.40 (*p* = 0.031) and nonsignificant χ^2^ difference from the configural models across weight status group (∆*χ*^2^ = 5.35 (*p* = 0.803)). However, all values of ∆CFI, ∆RMSEA, and ∆all values of ∆CFI, ∆RMSEA, and constrained models and items-intercept constrained models showed significant difference. 

Regarding the correlations between the WSSQ, PWSS, DASS-21, and BMI, Pearson correlation coefficients were all statistically significant. Specifically, the WSSQ (total score and each subscale score) was significantly correlated with PWSS. Both the WSSQ and the PWSS were significantly correlated with the DASS-21 (total score and each subscale score). However, BMI was not significantly correlated with the DASS-21 (total score and each subscale score) or the PWSS (Table 4).

## 4. Discussion

The present study was the first to examine the psychometric properties of the Thai-translated WSSQ and PWSS. Measurement invariance was performed to assess structure invariance across the gender and weight status subgroups of the Thai WSSQ and PWSS among Thai university students. The factorial invariance across different subgroups (i.e., gender and weight status) was obtained with adequate psychometric indicators (e.g., ∆CFI, ∆RMSEA, and ∆SRMR). Therefore, the Thai WSSQ and PWSS can adequately assess weight stigma with no difference in construct measurement across gender and weight status. Moreover, the scale items of Thai WSSQ and PWSS have revealed two-factor and single factor structures, respectively, with satisfactory psychometric indicators (e.g., factor loadings), similar to past studies [20,26,41]. The internal consistency of both the Thai WSSQ and PWSS had excellent reliability coefficients. In addition, both the Thai WSSQ and PWSS presented convergent validity through the association with other instruments related to psychological health (including depression, anxiety, and stress as measured by the DASS-21).

The findings showed that the Thai WSSQ presented satisfactory internal consistency illustrated by α = 0.83 for factor I and α = 0.86 for factor II. Consistent results were previously reported in an Iranian sample [2], with greater internal consistency in factor I (α = 0.91) than factor II (α = 0.87). The original version revealed similar results for the entire WSSQ questionnaire (α = 0.88), but factor II (α = 0.87) demonstrated a higher internal consistency than factor I (α = 0.81) (26). Likewise, Thai PWSS showed good internal consistency (α = 0.85). Similar results were demonstrated in a Malaysian study [20], with satisfactory internal consistency (α = 0.83), and the Chinese version revealed similar results with high internal consistency (α = 0.80–0.86) [18]. Additionally, the findings confirmed the two-dimensional structure of the WSSQ and unidimensional structure for the PWSS which was consistent with previous studies [20,26,41]. Therefore, the present results reveal that the Thai WSSQ and PWSS questionnaires had good reliability and construct validity among Thai university students. 

Similar to previous findings, the Thai WSSQ showed measurement invariance across gender (female vs. male) and weight status (non-overweight vs. overweight) [2,25]. The results illustrated that the factor structure of the WSSQ is similar across both the gender (female and male) and weight status (non-overweight and overweight) subgroups. Additionally, the Thai PWSS construct showed measurement invariance across gender and weight status. Measurement invariance of the PWSS in past studies has been acceptable across different populations (Hong Kong and Taiwan) [18]. To our knowledge, the present study was the first to examine measurement invariance of the PWSS across gender and weight status subgroups. In summary, both the Thai WSSQ and PWSS appropriately assess weight stigma across diverse groups of Thai university students. 

A positive significant correlation was shown between both questionnaires (i.e., Thai WSSQ and PWSS) and the DASS-21, which is similar to previous findings [2,18,19,20]. The past literature indicates that experience of weight stigma is related to, and may be mediated by, emotional distress (i.e., depression, anxiety, and stress symptoms) resulting in physiological problems such as disordered eating or lower quality of life [6,18,42]. Furthermore, our findings highlighted that there was a significant correlation between the Thai WSSQ and BMI. It is possible that individuals with an overweight experience increased weight stigma, potentially putting them at greater risk for mental health problems [41]. However, our findings showed the correlations between DASS-21 and PWSS were higher than the correlations with WSSQ. The previous study suggested that people were likely to misperceive their weight status, and it could impact on their physical health [18]. We hypothesized that the misperception of weight status might be more influential on psychological distress than self-devaluation and fear of enacted stigma.

Additionally, previous studies have reported that Thai university students may perceive their weight status incorrectly, which is also related to negative mental-health consequences [13,14]. As suggested by earlier studies, we hypothesized that there was a correlation between weight status and weight stigma, and this could relate to psychological distress among our participants. Furthermore, our results found that the PWSS scores had a stronger correlation with depression, anxiety, and stress than did the WSSQ scores, which contradicts one previous study, which reported that the WSSQ scores were more strongly correlated with DASS-21 scores than the PWSS scores [20]. Overall, the PWSS may be a strong predictor of psychological distress among Thai university students. In addition, we found the DASS-21 had a stronger correlation with the WSSQ fear of enacted stigma subscale than it did with the WSSQ self-devaluation subscale. We speculate that the fear of enacted stigma subscale might associate more with psychological distress (depression, anxiety, and stress) than the other subscale. Indeed, future studies should consider the effects of the fear of enacted stigma subscale on mental health problems more than the self-devaluation subscale.

Interestingly, our findings discovered the correlations between the WSSQ and PWSS were small in this present study. According to the literature [4,18], WSSQ and PWSS could assess different views of weight stigma. Thus, both psychometric measurements could examine independently the aspects of weight stigma and provide distinct negative health outcomes. Additionally, we found no correlations between DASS-21, BMI and PWSS and BMI in this present study. A Malaysian study suggested that the PWSS instrument was more likely to be assessing individuals with overweight and obesity than non-overweight [20]. We believe that there were no correlations between PWSS and BMI, this might be due to the fact that most of our participants were non-overweight (68.2%). Moreover, PWSS was significant associated with psychological distress (i.e., DASS-21). Accordingly, there is proven correlation between BMI and DASS-21.

According to the aforementioned, the WSSQ and PWSS could measure the different aspects of weight stigma. Nonetheless, further studies should examine the relationship between both WSSQ and PWSS with various significant factors (i.e., different populations or occupations) to be effective in relation to both psychometric measurements. Moreover, the future studies might include both the WSSQ and PWSS instruments which possess the stronger psychometric properties to assess a comprehensive view of weight stigma.

A strength of the present study is that it is the first study that has examined the Thai WSSQ and PWSS. These questionnaires show good psychometric properties, which support the previous evidence [20,26,41]. Moreover, this study extends testing invariance across different subgroups, with acceptable results in different genders and weight statuses. However, this study has several limitations that must be considered. First, our participants were recruited via convenience sampling. Therefore, this study may not be an accurate representation of the entire Thai population. Second, the Thai-translated WSSQ and PWSS were administered to Thai university students only. Future studies should assess and compare content validity in different age groups and occupations in Thailand. Third, it should be noted we conducted this study during the COVID-19 pandemic; therefore, results might present differently during normal conditions. For instance, a prior study showed that people experienced increased weight stigma through the media (e.g., internet memes) during the COVID-19 pandemic [43]. Therefore, prevalence rates of weight stigma experiences during the COVID-19 pandemic may be higher than before or after the pandemic. Fourth, the WSSQ and PWSS are self-report questionnaires. Participants might be influenced by social desirability or personal beliefs, which in turn may influence participants’ responses on the questionnaires. Lastly, the present results found many variables correlation which might restrict the generalizability of our findings. We suggest that future studies should further investigate the invariance of Thai WSSQ, PWSS and should be carefully and generally interpreted.

## 5. Conclusions

To summarize, this is the first study to assess the psychometric properties of the Thai-translated WSSQ and PWSS. The Thai WSSQ and PWSS are strong instruments for the evaluation of weight stigma. Moreover, this study indicated that both Thai instruments are invariant across the gender and weight status subgroups among Thai university students. Thus, the Thai WSSQ and PWSS can be measured as valid and reliable questionnaires to assess weight stigma among this population. This is of great significance for improving the assessment of weight stigma in Thailand. Both instruments may be crucial in clinical practice for healthcare professionals, and in decision-making and treatment planning to reduce weight stigma among Thai university students.

## Figures and Tables

**Table 1:** Item statistics and reliability for WSSQ and PWSS.

Items	Factor Loadings	Item-Total Correlation	Mean (SD)	Skewness	Kurtosis	α	ω
WSSQ						0.88	0.88
Factor I							
W1	0.60	0.56	2.92 (1.12)	−0.12	−0.70	0.83	0.83
W2	0.48	0.54	3.81 (0.93)	−1.10	1.48		
W3	0.75	0.67	3.20 (1.09)	−0.32	−0.45		
W4	0.75	0.69	3.10 (1.23)	−0.27	−0.93		
W5	0.61	0.51	3.25 (1.16)	−0.38	−0.63		
W6	0.78	0.61	3.08 (1.14)	−0.22	−0.77		
Factor II							
W7	0.59	0.45	3.14 (1.13)	−0.29	−0.53	0.86	0.86
W8	0.76	0.72	2.38 (1.12)	0.33	−0.78		
W9	0.70	0.61	2.98 (1.18)	−0.17	−0.84		
W10	0.82	0.76	2.46 (1.13)	0.26	−0.78		
W11	0.80	0.74	2.40 (1.13)	0.30	−0.84		
W12	0.64	0.66	2.07 (1.11)	0.68	−0.51		
PWS							
P1	0.63	0.58	0.23 (0.42)	1.30	−0.32	0.85	0.85
P2	0.67	0.61	0.20 (0.40)	1.51	0.27		
P3	0.58	0.54	0.10 (0.29)	2.77	5.69		
P4	0.68	0.62	0.15 (0.35)	2.02	2.10		
P5	0.65	0.60	0.13 (0.34)	2.21	2.88		
P6	0.56	0.51	0.18 (0.39)	1.66	0.76		
P7	0.60	0.56	0.11 (0.31)	2.54	4.47		
P8	0.61	0.56	0.16 (0.37)	1.87	1.51		
P9	0.48	0.46	0.05 (0.21)	4.27	16.24		
P10	0.58	0.53	0.21 (0.41)	1.42	0.02		

Note: Factor loadings were presented by standardized coefficients in the confirmatory factor analysis. WSSQ: Weight Self-Stigma Questionnaire; PWSS: Perceived Weight Stigmatization Scale; SD: Standard deviation; α: Cronbach alpha coefficient; ω: McDonald omega coefficient.

**Table 2:** The fit indices for the WSSQ and PWSS.

	WSSQ	PWSS
Fit indices		
χ^2^ (df)	271.20 (53)	44.88 (35)
*p*-value	<0.001	0.122
CFI	0.969	0.995
TLI	0.961	0.994
RMSEA	0.072	0.019
90% CI of RMSEA	0.063, 0.080	0.000, 0.033
SRMR	0.073	0.052

WSSQ: Weight Self-Stigma Questionnaire; PWSS: Perceived Weight Stigmatization Scale; CFI: Comparative fit index; TLI: Tucker-Lewis index; RMSEA: Root mean square error of approximation; SRMR: Standardized root mean square residual.

**Table 3:** Measurement invariance of WSSQ and PWS across gender (between female and male) and across weight status (between non-overweight and overweight) groups.

	WSSQ					PWSS				
	M1 (df = 106)	M2 (df = 116)	M3 (df = 126)	M2-M1 (df = 10)	M3-M2 (df = 10)	M1 (df = 70)	M2 (df = 79)	M3 (df = 88)	M2-M1 (df = 9)	M3-M2 (df = 9)
Gender										
χ^2^ (df) or ∆χ^2^ (∆df)	285.19	300.76	335.41	(15.57)	(34.65)	64.67	85.18	103.58	(20.51)	(18.40)
*p*-value	<0.001	<0.001	<0.001	0.113	0.0001	0.657	0.297	0.123	0.015	0.031
CFI or ∆CFI	0.975	0.974	0.970	(−0.001)	(−0.004)	1.000	0.997	0.993	(−0.003)	(−0.004)
RMSEA or ∆RMSEA	0.065	0.063	0.064	(−0.002)	(0.001)	0.000	0.014	0.021	(0.014)	(0.007)
SRMR or ∆SRMR	0.075	0.076	0.074	(0.001)	(−0.002)	0.064	0.069	0.066	(0.005)	(−0.003)
Weight status										
χ^2^ (df) or χ^2^ (∆df)	307.65	331.28	399.67	(23.63)	(68.39)	51.58	58.05	63.40	(6.47)	(5.35)
*p*-value	<0.001	<0.001	<0.001	0.009	<0.001	0.952	0.963	0.978	0.692	0.803
CFI or ∆CFI	0.969	0.967	0.958	(−0.002)	(−0.009)	1.000	1.000	1.000	(0.000)	(0.000)
RMSEA or ∆RMSEA	0.069	0.068	0.074	(−0.001)	(0.006)	0.000	0.000	0.000	(0.000)	(0.000)
SRMR or ∆SRMR	0.078	0.080	0.080	(0.002)	(0.000)	0.055	0.058	0.054	(0.003)	(−0.004)

WSSQ: Weight Self-Stigma Questionnaire; PWSS: Perceived Weight Stigmatization Scale; CFI: Comparative fit index; RMSEA: Root mean square error of approximation; SRMR: Standardized root mean square residual; M1: Configural model; M2: Model with factor loadings constrained equal across groups; M3: Model with both factor loadings and item intercepts constrained to be equal across groups.

**Table 4:** Correlation among WSSQ, PWSS, DASS-21, and BMI.

				r (*p*-Value)					
Variable	WSSQ (T)	WSSQ (S)	WSSQ (F)	DASS (T)	DASS (D)	DASS (A)	DASS (S)	PWSS	BMI
WSSQ (T)	--								
WSSQ (S)	0.90 (<0.001)	--							
WSSQ (F)	0.85 (<0.001)	0.55 (<0.001)	--						
DASS (T)	0.26 (<0.001)	0.20 (<0.001)	0.28 (<0.001)	--					
DASS (D)	0.23 (<0.001)	0.16 (<0.001)	0.26 (<0.001)	0.91 (<0.001) *	--				
DASS (A)	0.24 (<0.001)	0.18 (<0.001)	0.24 (<0.001)	0.90 (<0.001)	0.72 (<0.001)	--			
DASS (S)	0.25 (<0.001)	0.21 (<0.001)	0.27 (<0.001)	0.93 (<0.001)	0.77 (<0.001)	0.77 (<0.001)	--		
PWSS	0.29 (<0.001)	0.16 (<0.001)	0.37 (<0.001)	0.41 (<0.001)	0.36 (<0.001)	0.39 (<0.001)	0.38 (<0.001)	--	
BMI	0.32 (<0.001)	0.40 (<0.001)	0.14 (<0.001)	0.04 (0.258)	0.06 (0.115)	0.02 (0.518)	0.03 (0.384)	0.03 (0.386)	--

*p* < 0.001, * Using Spearman correlation. WSSQ(T): Weight Self-Stigma Questionnaire (Total score); WSSQ(S): Weight Self-Stigma Questionnaire (Self-devaluation subscale score); WSSQ(F): Weight Self-Stigma Questionnaire (Fear of enacted stigma subscale score); DASS(T): Depression Anxiety Stress Scale-21 (Total score); DASS(D): Depression Anxiety Stress Scale-21 (Depression subscale score); DASS(A): Depression Anxiety Stress Scale-21 (Anxiety subscale score); DASS(S): Depression Anxiety Stress Scale-21 (Stress subscale score); PWSS: Perceived Weight Stigmatization Scale; BMI: Body Mass Index.

## Data Availability

The datasets generated during and/or analyzed during the current study are available from the corresponding author on reasonable request.

## Abbreviations

WSSQ	Weight Self-Stigma Questionnaire
WSSQ(T)	Weight Self-Stigma Questionnaire (Total score)
WSSQ(S)	Weight Self-Stigma Questionnaire (Self-devaluation subscale score)
WSSQ(F)	Weight Self-Stigma Questionnaire (Fear of enacted stigma subscale score)
PWSS	Perceived Weight Stigmatization Scale
DASS-21	Depression Anxiety Stress Scale-21
DASS(T)	Depression Anxiety Stress Scale-21 (Total score)
DASS(D)	Depression Anxiety Stress Scale-21 (Depression subscale score)
DASS(A)	Depression Anxiety Stress Scale-21 (Anxiety subscale score)
DASS(S)	Depression Anxiety Stress Scale-21 (Stress subscale score)
BMI	Body Mass Index
SD	Standard deviation
α	Cronbach alpha coefficient
ω	McDonald omega coefficient
CFI	Comparative fit index
TLI	Tucker-Lewis index
RMSEA	Root mean square error of approximation
SRMR	Standardized root mean square residual.
M1	Configural model
M2	Model with factor loadings constrained equal across groups
M3	Model with both factor loadings and item intercepts constrained to be equal across groups.

## Appendix A

**Table A1:** Sample demographic (n = 801).

Variables	Mean (SD)	n (%)
Age (year)	20.69 (3.78)	801
Gender		
Male	--	265 (33.1)
Female	--	536 (66.9)
BMI (kg/m^2^)	21.90 (4.34)	--
Non-overweight	19.57 (1.88)	546 (68.16)
Overweight	26.89 (3.91)	255 (31.84)
Any condition or diseases		
Yes	--	66 (8.2)
No	--	733 (91.5)
Missing	--	2 (0.3)
Marital status		
Single	--	794 (99.1)
Married	--	5 (0.6)
Others	--	1 (0.1)
Missing	--	1 (0.1)
Grade		
Undergraduate	--	777 (97)
Postgraduate	--	11 (1.4)
Missing	--	13 (1.6)
WSSQ total score	32.71 (8.31)	--
Self-devaluation subscale score	19.36 (4.91)	--
Fear of enacted stigma subscale score	15.42 (5.23)	--
PWSS total score	1.50 (2.30)	--
DASS-21 total score	16.96 (11.65)	--
Depression subscale score	5.30 (4.27)	--
Anxiety subscale score	4.88 (4.06)	--
Stress subscale score	6.78 (4.41)	--

WSSQ: Weight Self-Stigma Questionnaire; PWSS: Perceived Weight Stigmatization Scale; DASS-21: Depression Anxiety Stress Scale-21; SD: Standard deviation.
